# Musculoskeletal Disorder Symptoms in Saudi Allied Dental Professionals: Is there an Underestimation of Related Occupational Risk Factors?

**DOI:** 10.3390/ijerph181910167

**Published:** 2021-09-27

**Authors:** Hadeel R. Bakhsh, Heba H. Bakhsh, Seham M. Alotaibi, Maha A. Abuzaid, Latefah A. Aloumi, Shoug F. Alorf

**Affiliations:** 1 Department of Rehabilitation, College of Health and Rehabilitation Sciences, Princess Nourah Bint Abdulrahman University, Riyadh 11671, Saudi Arabia; Hadeel@mail.net.sa (H.R.B.); 437000221@pnu.edu.sa (S.M.A.); 437000601@pnu.edu.sa (M.A.A.); 437000111@pnu.edu.sa (L.A.A.); shougfahad1998@gmail.com (S.F.A.); 2 College of Dentistry, Princess Nourah Bint Abdulrahman University, Riyadh 11671, Saudi Arabia

**Keywords:** musculoskeletal pain, prevalence, risk factors, allied dental, work-related injury, dental assistant, dental technician

## Abstract

This study aims to examine the prevalence of musculoskeletal disorder (MSD) symptoms in allied dental professionals (ADPs) in Saudi Arabia and risk factors for MSDs. The study also explores ADPs’ opinions and attitudes about the effect of MSD symptoms on the quality of life and potential mitigatory measures. This is a prospective cross-sectional study. Participants were 355 licensed dental staff (average age 33.6 ± 8.1 years, 69% women) working as assistants, lab technicians, radiology technicians, or sterilization technicians with at least six months’ work experience. The self-administrated questionnaire comprised socio-demographics, work-related factors, and items from the Nordic Musculoskeletal Questionnaire. Multivariate and univariate logistic regressions were conducted to investigate risk factors for MSD symptoms. Overall, 93% of ADPs complained of MSD symptoms in at least one body site in the past 12 months. Factors related to work conditions (e.g., ‘keeping an uncomfortable posture for long periods of time’, ‘lifting heavy objects’) and years of experience were proven to be significant risk factor for developing MSDs. The cohort of ADPs showed a high MSD symptoms prevalence. Efforts are imperative in addressing the risk factors of ergonomics and the workplace environment, and more rigorous studies are needed to further investigate risk factors.

## 1. Introduction

Musculoskeletal disorders (MSDs) represent a significant health problem worldwide with serious socio-economic consequences. The Global Burden Disease report in 2017 revealed that MSDs were the highest contributor to global disability—16% of all years lived with disability—with low back pain (LBP) being the single leading cause of disability since 1990 [1]. About a third of the worldwide population is affected by MSDs; thus, making them the most important cause of chronic disability that often results in the inability to work, absenteeism, a reduced quality of work, decreased job satisfaction, and increased incidence of work-related injuries [2,3,4,5].

It is well established in the literature that certain occupations are more susceptible to a higher prevalence of work-related MSDs than others, owing to the required skills and nature of work [6,7]. Occupations that involve frequently repeated forms of movements, as well as high physical demands in combination with psychosocial stress, are often associated with MSDs that regularly arise owing to specific work-tasks and awkward postures that workers undertake [8,9,10]. Therefore, dentistry has been the profession with the highest prevalence of MSDs compared to other healthcare workers [11]. Similar physical demands have also been observed among allied dental professionals (ADPs) (i.e., dental assistants, dental radiology practitioners, and dental lab technicians). Their work patterns consist of a static posture (standing or sitting), prolonged incorrect posture, and repeated gripping of small-sized instruments that demand precision [12].

This, in combination with other risk factors, have led to ADPs being one of the professions with the highest risk of developing MSDs alongside dentists and dental hygienists [12,13,14]. Such a high prevalence of MSDs among dentists and ADPs has been attributed to complex and several risk factors and it includes biomechanical, personal, and psychosocial components. Biomechanical risk factors involve repetitive tasks over a prolonged period of time, awkward and static postures [15]. The individual factors relate to the demography (e.g., sex, age, body mass index) and lifestyle (e.g., smoking, physical exercise). Lastly, psychosocial factors are subdivided in three categories that refer to work organizational factors (e.g., working hours and load, mental demand), external work environment relating to responsibilities and duties towards family and friends, and the individual’s characteristics (e.g., culture, job satisfaction, social class, etc.).

Numerous studies have demonstrated a high prevalence of MSDs among dentists and ADPs worldwide ranging from 62 to 96% [16,17,18]. In Saudi Arabia, the prevalence of MSDs—specifically LBP—has been investigated in many recent studies [19,20,21]. However, most of these studies were looking at specific cohorts and occupations rather than the whole population of Saudi Arabia. For example, the prevalence of MSDs was recorded at 80% among nurses [20], 73.9% in other healthcare staff [11], 68% among female secondary school teachers [22], and 57.3% among male high school teachers [23]. Additionally, a recent systematic review found that LBP prevalence in Saudi Arabia ranged between 53.2% and 79.2% [19,21]. So far, studies on the prevalence of MSDs in the dental field in Saudi Arabia have mainly been focused on dentists and dental students [24,25,26,27]. Although a significant difference has been found between the prevalence of MSDs between dentists and ADPs [28,29,30], studies investigating the prevalence of MSDs among ADPs in Saudi Arabia are scarce. Only two studies in the last decade have investigated the prevalence in this subgroup of professionals in the dental field [31,32]. The two studies reported an MSD prevalence of 59%–73% among ADPs in the past 12 months. However, these studies had several methodological limitations, including a small sample size and lack of homogeneity in the sample (e.g., lack of appropriate representation of ADPS with the majority being dental specialists) [31,32]. 

Therefore, the main aim of the current study is to evaluate the prevalence of MSD symptoms in ADPs in different work settings (private, government, or academic) and to investigate potential associations with different sociodemographic and work-related variables. A secondary aim is to explore the ADPs’ opinions and attitudes about the effect of MSD symptoms on the quality of life and their perspective on potential ways to mitigate the issue to help policymakers obtain new insights on unexplored factors for future research and consideration.

## 2. Materials and Methods

### 2.1. Study Design and Sample Size

**Figure 1:**
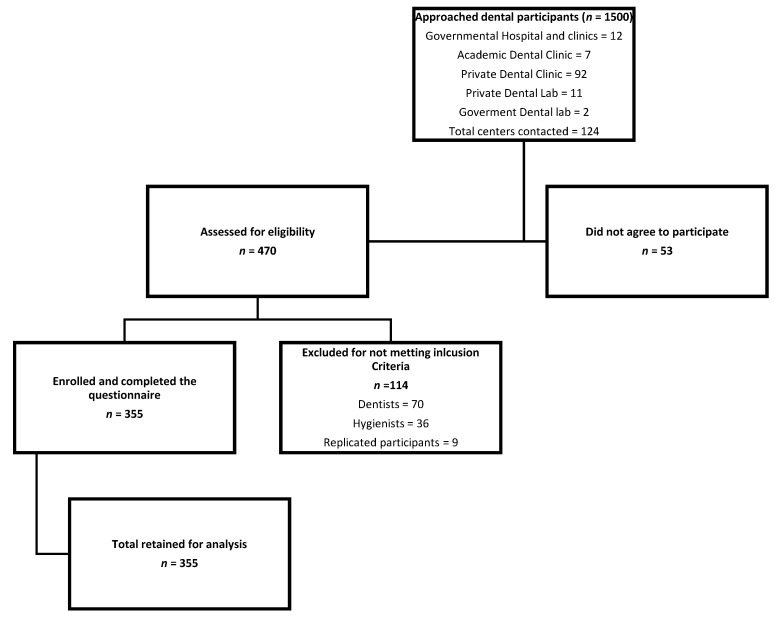
Flowchart of participant recruitment process.

This was a prospective cross-sectional analytical study conducted in a convenience sample of 129 dental facilities in Saudi Arabia. Randomization of the sample was not possible as it was difficult to ascertain the representation of all the target categories of ADPs in the randomly selected centres (e.g., some centres would not have a lab; thus, lab technicians would be left out). An estimated total of 1500 ADPs was approached between August and December 2020. Approached centres agreed to participate by allowing the authors or the supervisors/managers to approach and recruit a convenience sample of all available participants during regular working hours. Participants were given either a link to a web-based questionnaire and/or hard copies to complete. Figure 1 illustrates the recruitment process of participants. The primary outcome of this study was the 12-month prevalence of MSD symptoms in ADPs; therefore, to extrapolate the study results, sample size was calculated using the following formula: *n* = *Z*^2^*P*(1 − *P*)/*d*^2^ (*Z* = *Z* statistics for confidence level, *P* = expected prevalence or proportion, *d* = precision). The confidence interval was set at 95% (*Z* = 1.95), precision 5% (*d* = 0.05), and prevalence was expected at midpoint of 66% (*P* = 0.66) based on previous studies [21,32], Thus, the minimum number of study subjects required for a representative sample of the studied population was determined as 345 [33,34,35]. 

### 2.2. Participants

Participants were eligible if they were licensed and working as a dental assistant, dental lab technician, dental radiology technician, or dental sterilization technician with at least six months of work experience. Participants were excluded if they had less than six months of work experience, systemic disorders, history of musculoskeletal trauma, were pregnant women, or were students or interns. Students/interns were excluded owing to their lack of practical experience and different nature of responsibilities as observers in the clinical setting. Respondents with missing important information (e.g., gender) and incomplete questionnaires were excluded. This study was performed in line with the principles of the Declaration of Helsinki. Approval was granted by the Ethics Committee of Princess Nourah Bint Abdulrahman University (IRB log number: 19-0092; IRB Registration with KACST, KSA H-01-R-059) and was funded through the project number PNU-DRI-RI-20-001.

### 2.3. Outcome Measure 

To evaluate the factors shaping the presence of MSD symptoms, a self-administrated questionnaire was used, and it consisted of three main parts:

The first part contained basic questions on socio-demographic characteristics of participants (e.g., age, body mass index (BMI)).

The second part contained work-related questions (e.g., professional group, experience in years, average weekly working time, dental practice setting, knowledge, and application of ergonomic posture). This part was developed through extensive literature review and discussion among the research team and experts to ensure the validity and relevance of the questions [3,24,36].

The third part—the Nordic Musculoskeletal Questionnaire (NMQ)—was used both in English and the validated Arabic language version in the same survey to cater for language preference (no cross-cultural issues previously reported in using the translated version) [3,24,36,37]. The NMQ consisted of 11 questions addressing the onset and prevalence of pain (lifetime, annual, weekly) in nine body areas (neck, shoulders, upper back, elbow, wrist/hand, low back, hip/thigh, knee, ankle/foot) with binary choice questions (Yes/No) and was accompanied by an anatomical diagram showing the specified sites. 

Moreover, the NMQ evaluated the need of medical care, use of medications, sick leave due to pain experienced, and changes to the personal routines of activities of daily living. The NMQ was selected as an appropriate tool for our cohort study as it is a globally used, well-documented, valid, and reliable tool for assessing MSD symptoms in different occupations [38]. To ensure applicability and clarity in English and Arabic, the questionnaire was pilot tested among 10 individuals within the target group. Consequently, re-evaluation was done, and adjustments were performed. The Arabic NMQ was reported to have a very good reliability with Kappa coefficient values of >0.88 [36].

Finally, the questionnaire had a supplemental optional open-ended part: two non-mandatory open-ended questions were added to obtain more insight into the effect of MSD symptoms on daily life and suggestions to help solve the problem. “Please tell us how your musculoskeletal pain is affecting your quality of life” and “Please share with us any suggestions or comments that would help in improving the quality of life and working environment”. The questions aimed to open room for more ideas to explore effect of MSD symptoms on quality of life (QoL). The participants could use Arabic or English in their answers to accommodate for language barriers and allow participants to use the preferred language to communicate their ideas clearly. 

### 2.4. Data Collection Procedure 

Participants were approached via different recruitment strategies. The team searched well-known dental facilities located in and outside of Riyadh. Facilities located in Riyadh were approached by the research team in person or via phone call and spoke to the management head to arrange the distribution of the survey among ADPs. Facilities located outside of Riyadh were approached via phone call by one of the research team members. The data were collected by four occupational therapists (co-authors), who were not affiliated to any of the approached hospitals/centres. Furthermore, the authors shared the survey with dental professionals and their networks with information distributed through dental clinics across Saudi Arabia. Prior to enrolment in the study, the authors provided an explanation and information about the purpose of the study; participants then anonymously completed and signed an informed consent form. Participants were instructed to complete the questionnaire either through the web-based link (Google Forms) or the hard copy. The questionnaire took approximately 10 min to complete.

### 2.5. Risk Factors 

Work characteristics reported by the participants were included to investigate their possible effects on MSDs. Variables included work setting, occupation (e.g., dental assistant), years of experience, working hours (average hours worked per week), and type of physical movement performed during work (e.g., bending, rotating). 

Health-related variables included age, sex, height, weight, and BMI interpreted with cut-offs identified by the World Health Organization [39].

### 2.6. Statistical Analysis 

Descriptive statistics were used to summarise data. For categorical variables, frequencies and percentages were calculated. For continuous variables, mean ± SD was used. Univariate and multivariate logistic regressions were conducted to assess the association between risk factors related to MSD symptoms in ADPs. The univariate model was run to identify factors that showed an effect without controlling for the effect of other factors. A backward stepwise approach for model building was utilized to decide on the variables to be included in the final model [40,41]. The final multivariate logistic model included all variables with a potential association with MSD symptoms. The association of the risk factors with MSD symptoms was assessed at the 5% significance level (variables were considered significant at *p* < 0.05). SPSS statistical package version 26 was used for all analyses [42].

### 2.7. Qualitative Analysis 

Phenomenological analysis was used to develop patterns, categories, and themes to obtain an understanding of individuals’ experience of the effect of MSD symptoms on QoL and ways to mitigate the problem from the participants’ point of view [43]. Arabic answers were translated to English by one of the authors (HHB). Transcripts were imported to QSR International’s NVivo 13 qualitative data analysis software and was analysed manually in the software [44]. An inductive method was followed to generate codes from the participant’s own words or that which reflected concepts they were referring to. Codes were grouped to develop categories related to the phenomenon under investigation [43]. Themes then emerged by combining related categories and interpreting them to highlight the participants’ experiences. The frequency of each theme was reported to give a sense of significance to the subject reported.

### 2.8. Quality Assurance

Data were collected during a limited time frame and extracted into Excel and SPSS software by one author (SA). Prior to analyses, data were checked twice by the second author (HHB). Qualitative analysis was conducted by one author (HHB). All authors were well acquainted with the data and preparation of the manuscript.

## 3. Results

### 3.1. Socio-Demographic Characteristics

A total of 470 individuals agreed to participate and completed the questionnaires at a response rate of 31%, of which 115 were excluded as they did not meet the inclusion criteria and the presence of replicas of email accounts was reported. A total of 355 participants were included in the final analysis (Figure 1). Of these, women accounted for 69% (*n* = 245), participants’ average age was 33.6 ± 8.1 years, and 44% had a normal BMI. Dental assistants accounted for 70% of the participants and, overall, 41% participants worked 40 to 45 h weekly. Socio-demographic and work-related characteristics of the participants are presented in Table 1.

### 3.2. Prevalence 

**Figure 2:**
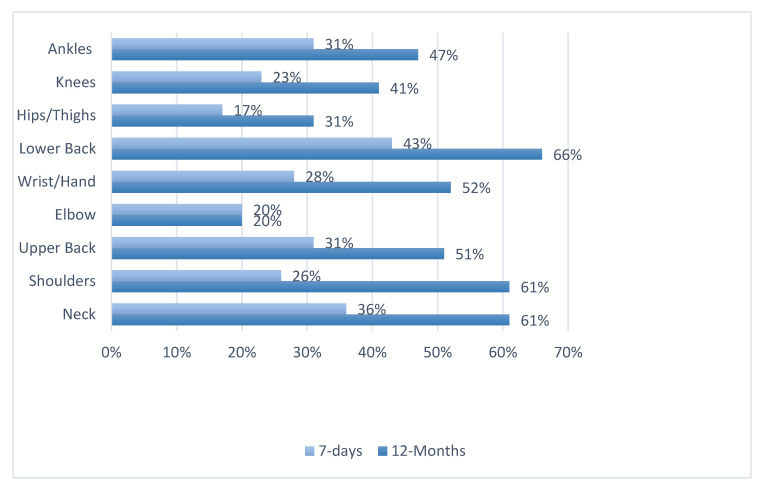
Prevalence of reported musculoskeletal disorder (MSD) symptom complaints in different body parts over 7-day and 12-month periods for all allied dental practitioner (ADP) occupations (*n* = 355).

The percentage of ADPs complaining of MSD symptoms in at least one body site in the past 12 months was 93%. The highest prevalence belonged to the lower back (66%), shoulders (61%), and neck (61%) (Figure 2), whereas Table 2 demonstrates the breakdown of prevalence MSD symptoms among each body part for each of the different occupations. The most typical physical movements for ADPs (Table 3) were standing for a long time (75%), repetitive hand and wrist movement (60%), and an uncomfortable posture (50%). Moreover, the highest prevalence of ADPs visiting a physician for an MSD symptom complaint was for the LBP (28%), neck (24%), and shoulders (22%), for which the prevalence of taking medication for pain was also the highest at 38%, 31%, and 30%, respectively; the most affected were dental radiology technicians followed by dental sterilization technicians (Table 4). In this study sample, 39% participants reported having received information about ergonomics and proper positioning, and only 18% reported having a work-related injury. Around half of the participants (51%) reported poor sleep due to MSD symptoms (51%) and 52% reported pain affecting activities of daily living (21% were unsure).

### 3.3. Associated Risk Factors

The frequency distribution of MSD symptoms for the nine body regions were almost symmetrical (mean 4.31 ± 2.5). The univariate analysis showed no significance for the following variables: sex, age, height, weight, BMI, dental setting, and working hours (*p* > 0.05). The multivariate analysis showed that the following factors increased the risk of MSDs (in a sequential order of effect): ‘Keeping an uncomfortable posture for long periods of time’ (*p* = 0.000), ‘lifting heavy objects’ (*p* = 0.004), and ‘years of experience/working’ (*p* = 0.019). Age closely associated with years of experience (*r* = 0.75). In contrast, being a ‘dental lab technician’ lowered the risk for MSD symptoms (*p* = 0.005, standardized coefficient beta (β) = −2.839). Those with a very long duration of work experience (more than 15 years) tended to be less at risk than those with 10 to 15 years of experience; this fact is evident from the general positive coefficient for years of experience (β = 0.227), but a negative coefficient for age (β = −0.150) in the first stepwise analysis.

In the second stepwise analysis, “years of experience” when modelled by dummy variables indicated that those with 10 to 15 years of experience had a significantly higher chance of MSD symptoms compared to other categories. Finally, an analysis of residuals showed no divergence from normality and, hence, the regression model was an acceptable model for this analysis.

### 3.4. Participants’ Opinions

**Figure 3:**
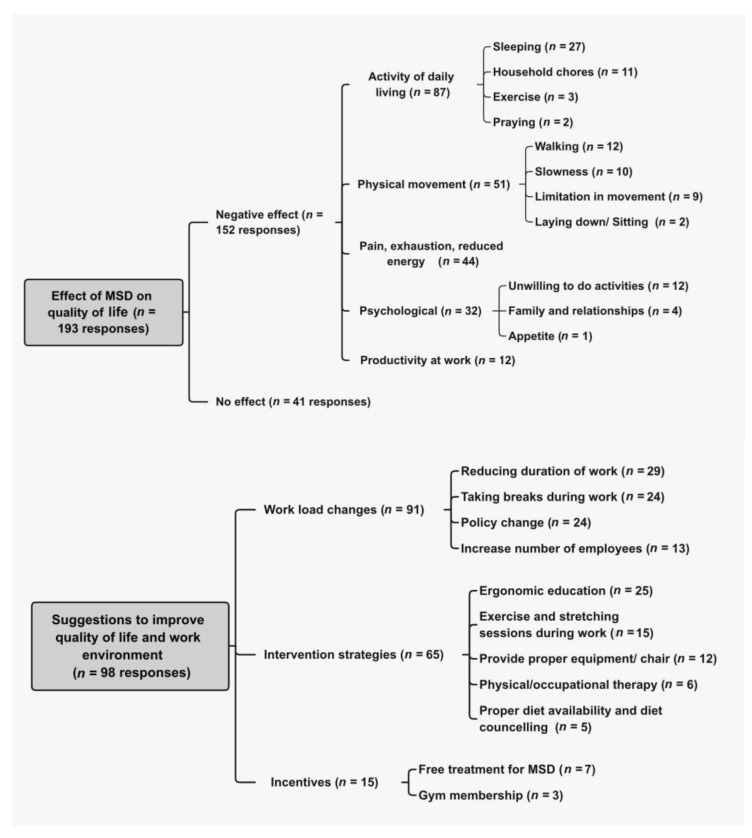
Participants’ responses with emerging themes for the two open-ended questions.

Participants’ responses for the open-ended questions were coded and presented into themes, as demonstrated in Figure 3. When asked about the effect of MSD symptoms on the QoL, 193 respondents answered the question; 41 (21%) reported no impact of MSD symptoms on the QoL, and the remaining 152 (79%) reported adverse effects. Negative effects on activities of daily living included difficulties in daily activities, sleeping, household chores, exercise, praying, and adverse effects on appetite; as one participant reported, “It affects negatively, I find it difficult to sleep from pain, prayer, and all my activities, and it also reduced my productivity at work”. 

Physical movement hindering was also frequently mentioned as slowness or pain/limitation while walking, sitting, and laying down; one participant responded that she “Can’t finish work on time.” Pain, a lack of energy, and exhaustion were reported by 44 participants as described by one participant “I feel exhausted and tired after work, and I avoid making any effort after it”. Negative psychological effects of MSD symptoms were reported by 32 participants; “Feeling pain and discomfort in different parts of my body stresses me so much,” another writing “Sometimes I feel tired and depressed.”

When asked about suggestions on how to reduce MSD symptoms and their burden, 137 participants replied with answers that represented one of the following themes: workload reduction, interventions, and incentives. Reducing the workload was among the most frequent demand from participants. Suggestions included increasing the number of employees “I really hope that two dental assistants are hired and work with each doctor in order to relieve the work pressure.”, enabling regular breaks “I suggest that in every Dental clinic, there would be at least 30 min to 1 h break to relax and stretch after so many patients.”, and reducing the duration of clinical work.

Participants suggested some interventions, including educating staff about proper ergonomics, encouraging stretching and exercise during work, providing proper equipment, providing physical and occupational therapy sessions, and an appropriate diet; “Exercising helps a lot to alleviate the problem, but it needs time”. Several participants complained about the provision of poor chairs “I work on an inappropriate chair, and I complained more than once, but it was not replaced, the chair is very important.” While some asked for policy changes in favour of general health and comfort of the workers, such as flexible hours and alternating schedules to allow for time to rest; some simply suggested being fair with everyone “Give enough time for every procedure, Sterilization staff should not be a dental assistant (female)”. 

Some workers complained of how they were obligated to work long hours because of their status as expatriates under the governmental labour law. Finally, some participants suggested incentives such as a free gym membership or free physical or occupational therapy sessions. The low response to these two questions is because, firstly, they were optional questions. Secondly, the practitioners lead a busy schedule reflected by the long working hours (40+ hours per week). 

## 4. Discussion

While the prevalence of work-related MSDs in the dental field and associated risk factors have been broadly investigated, essential gaps exist in exploring specific dental professions. The present study focused on investigating the prevalence of MSD symptoms in a representative sample of Saudi ADPs and the potential associations with different sociodemographic and work-related variables. Furthermore, this study shed more light on this group of dental professionals and their perception about work-related MSD symptoms as they are often neglected in prevalence studies. 

### 4.1. Prevalence

The current study showed a high prevalence of annual reported MSD symptom complaints among ADPs. In total, 93% of the sample reported MSD symptoms of at least one body site during the past 12 months. Other studies showed that the 12-month prevalence of MSD symptoms of any body site for ADPs ranged between 44.9% and 97.5% [28,29,45,46]. Very few studies have assessed MSDs among ADPs in Saudi Arabia. AlGhadir et al. [32] reported a high prevalence of MSDs in ADPs in Saudi Arabia with at least 85% of the participants reporting the development of some MSD following their enrolment in the dental profession. However, the sample size was small (*n* = 146), with only 5% representing ADPs [32]. Another study by Alwazzan et al. [31] involved only MSDs of the back and neck among dental professionals in Riyadh, Saudi Arabia, and had a higher sample size of ADPs; they reported that 37.5% dental assistants suffered from neck pain and 63.8% from back pain at some time in their lives. Meanwhile, 63% of dental technicians had neck pain and 72.4% had back pain.

In this study, the body sites with the highest prevalence of MSD symptoms were lower back, neck, shoulders, and upper back (66%, 61%, 61%, and 50%, respectively). Among dentists in Saudi Arabia, lower back, neck, and shoulders were also the most prevalent body sites for MSDs [25,47]. These findings match the numbers reported among German [45], Thai [29], and Sudanese ADP populations [46]. However, in a study by Ohlendorf et al. [45], dental assistants reported a higher prevalence of MSDs of the neck (85%) and shoulders (70%) compared to our results, but similar percentages for the lower back (60%) and upper back (48%). Moreover, in a sample of dental assistants from Thailand, the prevalence rates of MSDs of the shoulders, neck, and lower back were 72.2%, 70.3%, and 50.6%, respectively [29]. Lastly, Osamn’s study with a sample of dentists and ADPs in Khartoum, Sudan, reported prevalence rates of 58.4% for the shoulders, 54% for the neck, and 75.2% for the back [46].

In Western countries, a meta-analysis of MSDs among dental professionals (41 studies included) found neck, lower back, and upper back to be the most prevalent among body sites with pooled percentages of 58.5%, 56.4%, and 41.1%, respectively [48]. 

To better understand the study results, the data were compared to MSD prevalence in the general Saudi population to demonstrate that the prevalence of LBP in ADP is comparable to the general population. A systematic review of LBP prevalence in Saudi Arabia (with seven cross-sectional studies) found a prevalence and pattern ranging from 53.2% to 79.17% [19,21]. This indicates that ADP prevalence of LBP is similar to the range of prevalence in the general population in Saudi Arabia. Furthermore, a recent study by Alnaami et al. [11] on the prevalence of LBP in various healthcare professionals (including dentists) in Southern Saudi Arabia showed an overall prevalence of 73.9% (*n* = 740).

### 4.2. Associated Risk Factors

Previous studies documented the influence of several factors such as age, gender, ergonomics, a lack of job satisfaction, being overweight or obese, lack of physical activity, and stress on aggravating MSD intensity. Identifying these factors is essential as it allows for designing a suitable prevention strategy [49,50]. The results of this study demonstrated no significant association between MSD symptoms and sex, BMI, height, weight, working hours, or work setting.

#### 4.2.1. Type of Physical Movement

The results of this study found that keeping an uncomfortable posture for long periods of time and lifting heavy objects increased the risk of MSD symptoms among ADPs for the four predominant pain areas and during the three investigated time periods. Multiple studies linked an abnormal and inconvenient working posture with back and neck pain [51,52,53]. Samat et al. [28] reported that the odds of having back pain were 3.5 times more for dentists and ADPs with a compromised posture compared to those with an acceptable working posture. Additionally, Alghadir et al.’s [32] study in Saudi Arabia with a sample of dental personnel, including dental auxiliaries, reported that the respondents attributed their MSDs to frequent strenuous back positions, repetitive hand and shoulder movement, a high job demand, and high exertion [32]. Moreover, Lietz et al. [48] identified an awkward posture while working as a potential risk factor for MSDs among dental professionals among other factors, including a high patient volume, administrative work, vibration, and repetition [48]. 

Regarding working seated or standing, studies did not find a difference in MSD risk between sitting and standing [12]. Moreover, Samat et al. found that repetitive movement, prolonged sitting, and excessive movement were not associated with an increased risk of MSDs [28].

#### 4.2.2. Age and Years of Experience

The findings of this study showed that ADPs with long experience reported MSD symptoms less than those with 10–15 years of experience. This could possibly be due to more experience in managing the workload and experience in the best practices to reduce work pressure. It could also be hypothesized that practitioners with very long experience have a managerial workload and less physical demand compared to mid-career workers. However, responsibility types were not investigated in this study. Al-Gunaid et al. [25] found significant correlations between shoulders, upper back, and lower back pain with years of experience in dentists. It was reported that less experienced dentists were more likely to have MSD symptoms than their more experienced counterparts as experienced dentists are probably better at adjusting their working position and techniques to avoid musculoskeletal problems or they simply developed coping strategies to deal with the pain [25]. 

Meanwhile, age was found not to be a risk factor for MSDs by Al Wazzan and Samat et al. [28,31]. Furthermore, age was found to be a significant factor for back pain in a study by Leggat et al. [54] which reported that back pain was more common among younger dentists. Such findings could be attributed to work inexperience and inadequate knowledge in dental procedures. Knowledge about the scope of ergonomics and prophylaxis as well as health and safety measures at the workplace are important to prevent the risk of developing back pain.

#### 4.2.3. Occupation

In this study, dental lab technicians had significantly lower MSD symptoms compared to dental assistants, radiology, and dental sterilization technicians. This might be due to the working nature of each job as the lab technicians do not work on patients, work seated most of the time, and are among the least in lifting and pushing heavy objects (Table 2). Moreover, dental technicians were also found to be lowest in visiting a physician and taking pain medication for most body areas compared to other ADPs (Table 3). 

Looking at dental assistants, their work side-by-side with dentists by assisting them, requiring them to adapt to the ideal position for the dentist requiring standing, keeping an uncomfortable position. They also manage cleaning the dental clinic, bringing requited instruments and material, and managing the administrative work. Sterilization technicians in this sample were found to stand for long periods of time, are the most likely to lift and push heavy objects, and perform bending motions which places them at a higher risk for MSD. Lastly, radiology technicians stand, perform rotational movements and repetitive hand and wrist movements more frequently (Table 2). Similar findings were seen in a study by Šćepanović et al. [12], where 82.6% of general dentists had MSD, 75% of dental specialists, 66.7% of dental assistants, including 33.3% of the dental technician sample; however, no explanations were offered for the difference observed. 

On the other hand, Samat et al. [28] found that dental technicians had the highest prevalence of back pain (52%) followed by dental assistants, dental nurses and dentists (48%, 44.8%, 28%). Samat et al. attributed the difference to the continuous abnormal posture while sitting that strains the spine and the supporting tissues, as well as the higher demand of cosmetic lab fabricated prosthesis and an accompanying increased workload. This difference might be due to limiting their study to back pain only, while this study covered all body sites and included four different body sites in the regression analysis. 

### 4.3. Non-Significant Risk Factors 

Interestingly, evidence recognizing gender as a risk factor for developing MSDs exists in literature with females being more prone to MSDs [21,32,45,55,56]. Yet, in this study, gender-related differences were not statistically significant, which was consistent with studies by Šagát et al. [57] and Hashem et al. [58]. The real impact of gender as a contributing factor in MSDs among ADPs needs to be clarified in future research. In comparison to studies with dentists of both sexes, high percentages of MSDs in dental healthcare professionals prevail, independent of the gender. 

Furthermore, this study identified no significant association of MSD symptoms with weight, height, or BMI. This contradicts some studies that found an increased BMI to be a risk factor for developing MSDs. However, there is minimal evidence to suggest that these individual factors have an additive effect when combined with physical work-related factors in the contribution to MSDs. Thus, these disorders may occur in the absence of any workplace exposures or events. 

There are several issues in establishing the relative influence of physical and psychosocial factors. One concern is that psychosocial factors are usually measured at the individual level, whereas physical factors are often measured at the group level (e.g., measuring a job or task) and the methods used often lack accuracy and precision [2]. Second, it is hard to find objective measures for various aspects of the psychosocial work environment while those that measure the physical environment are more easily accessible [59]. Moreover, Alwazzan et al. [31] argued that the nature and type of work dental assistants perform is less stressful, and induces less strain on the spine in comparison to dentists. They also mentioned that tasks assigned to dental assistants allow for greater mobility and frequent postural changes throughout the working day compared to dentists and hygienists [31]. 

### 4.4. Participants’ Suggestions for Reducing Burden of MSD Symptoms

#### 4.4.1. Workload Reduction

In an open-ended question, the most reported suggestion from the participants to reduce MSD symptoms was a workload reduction; this included reducing the session duration and workload, enabling breaks, and increasing the number of employees. The suggestion determined by the participants was consistent with published studies that recommended the inclusion of breaks during the working hours as a strategy to reduce MSD symptoms among dental practitioners [12,29,60]. Osman found that taking breaks between appointments was significantly associated with a reduced prevalence of pain [46]. Almas et al. [61] found that increased weekly working hours resulted in an increased prevalence of back pain. Similarly, Al-Mohrej et al. [26] reported that LBP was related to the duration the dentist spends with patients. 

However, in this study, weekly working hours were not associated with increased MSD symptoms. Additionally, there was no difference in the status of working hours in the sample, as 95% of the sample worked full time (35–45 h per week); the maximum working hours set by the Ministry of Human Resources and Social Development is 48 h per week. Currently, in Saudi Arabia, working part time is not an easily attainable option for healthcare professionals, resulting in the low percentage (5%) of ADPs working less than 35 h per week. Aspects such as working less than 35 h per week and the specific nature of work and movement involved could not be investigated. 

#### 4.4.2. Ergonomics Education

The most requested intervention strategy by participants was a proper ergonomics education as an intervention to MSD symptoms. In the surveyed sample, 58% had knowledge about proper ergonomics and posture, while 42% did not or were not sure. Only 5% reported following ergonomic positions all the time and 17% rarely/never followed it. This might be due to being influenced by the questions asking about knowledge of proper ergonomics for the job.

According to a systematic review by Roll et al. [62], when ergonomic training is implemented in practice, it seems to decrease musculoskeletal pain effectively, which shows the importance of education, as well as the potential of symptom exacerbation with a lack of education [62]. Furthermore, equipment that promotes ergonomic techniques have the potential to decrease the prevalence of musculoskeletal pain and the consequent absence from work [62,63].

Of the ergonomics interventions investigated, moderate to moderately strong evidence supported the use of lighter/wider hand instruments, favourable positioning, and the introduction of microbreaks during lengthy procedures to reduce upper limb MSDs [64]. As an example, decreasing the angle of neck flexion required during dental procedures is associated with less trapezius muscle activation, resulting in less fatigue [65]. Similarly, decreasing the load in the upper limb by supporting the limb weight requires less upper limb muscle activity and reduce fatigue in these muscles [66]. A reduction in muscular load has been associated with less musculoskeletal pain and discomfort in the upper limbs [64]. 

Interestingly, a few participants’ responses included asking for proper equipment, specifically chairs. Alghadir et al. found that revolving chairs were associated with higher neck and shoulder MSDs, while chairs with an arm rest resulted in less reported pain compared to chairs without an arm rest [32]. However, other studies found that pain intensity and MSD prevalence were not associated with the type of chair; nevertheless, pain-related disability was significantly reduced with the use of movable and rotating chairs [28,46]. 

More studies are needed to evaluate the posture and ergonomics for ADPs, specifically dental assistants, because of the dental setting and the fact that patient position is set to the dentist’s comfort, resulting in dental assistants having to adapt to assist the dentist properly. Additionally, ergonomics education should be implemented and emphasized in ADPs’ education despite their short training period. Creating theoretical and practical knowledge would assist in reducing the prevalence and related burden of MSDs among ADPs. The positive impacts of such educational training on MSD prevalence should be analysed in future studies [45]. 

#### 4.4.3. Exercise 

Fifteen participants suggested exercises and stretching as means of preventing and tackling MSDs. Stretching/yoga and chairside stretching were reported to be a preventive factor for work-related MSDs for dentists and dental hygienists [56,67]. Stretching keeps muscles flexible and reduces tissue restrictions. Abundant evidence is available, highlighting exercise as a preventive measure and treatment for MSDs among dental professionals [12,26,58,63,64]. 

### 4.5. Strength, Limitations, and Future Recommendations

The strength of this study was the relatively large sample size of allied dental professionals in Saudi Arabia compared to previously published studies of the same population—which had issues of a small sample size, lack of homogeneity, or targeting dentists, hygienists, or dental students. Moreover, our study contained a qualitative analysis of the participants’ opinions on the effect of MSD symptoms on their quality of life and suggestions to improve their work performance. To our knowledge, and up to date, no previous study in Saudi Arabia conducted such an analysis. 

However, this study suffered from a few limitations. Firstly, despite the large number of approached subjects (1500), the response rate was low (24%) despite using two methods to collect data (online and hardcopies), which might be attributed to the busy schedules of the ADP as most participants (41%) worked 40–45 h and more than 45 h (34%); thus, after such long hours of work, one may forget to complete the survey or may not be interested to do so. The external validity of the study may be affected and limited due to the nature of convenience and accidental sampling of participants who are easy to access, which in turn could bias the prevalence results. Future studies should use random sampling from different locations in Saudi Arabia. 

On the other hand, the variety of dental centres and work environments spread over a large geographical region of Saudi Arabia may have compensated for this shortcoming. Moreover, this study may have been subjected to volunteer bias, since a large proportion of the study participants were female, who tend to be more sensitive to pain compared to males, and/or may be highly motivated to participate in this study and contribute to the particular issue [45]. Moreover, the type of convenient sampling of participants could lead to a biased prevalence and, thus, limit the external validity of the study. Another possible limitation was the time of data collection coinciding with the current pandemic of COVID-19, which may have potentially affected the prevalence of self-reported MSD due to the lockdown, curfew, and limited working hours decreed in Saudi Arabia during 2020. In this respect, it is necessary to recognize that some authors consider that pain recall is not entirely reliable. Lastly, although the use of medication and physician visits was utilized as an indication of pain severity and the effect of MSD on ADL, our study did not directly evaluate pain severity; thus, we recommend that future studies address this factor.

Therefore, it is recommended that the ergonomics of ADPs’ work environment be improved. This can be achieved by training courses covering occupational health, ergonomics, workplace organisation, and psychosocial coping skills should be offered to ADPs. Thus, further studies on the ergonomics in relation to ADPs are encouraged [26].

## 5. Conclusions

In summary, ADPs are considered among the professions with the highest risk of developing MSD symptoms alongside dentists and dental hygienists due to the required working skills and tasks. The current study showed a high prevalence of MSD symptoms in a large cohort of ADPs in Saudi Arabia. The most affected body areas were lower back, neck, shoulders, and upper back. It is imperative to dedicate efforts in addressing the risk factor of ergonomics and the workplace environment. A comparison with the existing literature had significant limitations; thus, more rigorous studies are needed to investigate the risk factors of developing MSD symptoms among ADPs.

## Figures and Tables

**Table 1:** Study Participants’ Characteristics *n* = 355.

Sociodemographic Characteristics	*n* (%) or Mean ± SD
Gender, *n* (%)	
Female	245 (69%)
Male	110 (31%)
Age, mean ± SD	33.6 ± 8.1
Height, mean ± SD	162.7 ± 9.8
Weight, mean ± SD	69.8 ± 19.5
BMI, mean ± SD	26.2 ± 5.9
Underweight (<18.5 kg/m^2^)	17 (5%)
Normal (18.5–25 kg/m^2^)	159 (44%)
Overweight (25–29.9 kg/m^2^)	105 (29%)
Obese (>30 kg/m^2^)	75 (21%)
Occupational characteristics, *n* (%)	
Occupation	
Dental assistant	249 (70.1%)
Dental lab technician	64 (18%)
Dental sterilization technician	32 (9%)
Dental radiology technician	10 (2.8%)
Work setting	
Governmental hospital	106 (29.9%)
Governmental dental centre	76 (21.4%)
Academic dental clinic	45 (12.7%)
Private dental clinic	105 (29.6%)
Private dental lab	17 (4.8%)
Other	6 (1.7%)
Years of experience	
0.5–1 year	38 (10.7%)
1–3 years	84 (23.7%)
3–5 years	57 (16%)
5–10 years	80 (22.5%)
10–15 years	53 (14.9%)
15–20 years	24 (6.8%)
More Than 20 Years	19 (5.3%)
Working hours	
Less than 35 hours	17 (4.8%)
35–40 hours	73 (20.6%)
40–45 hours	144 (40.6%)
More than 45 hours	121 (34%)
Physical demands during working hours	
Less than 30%	25 (7%)
30–50%	49 (14%)
50–75%	75 (21%)
More than 75%	109 (31%)
100%	97 (27%)
Ergonomic knowledge, *n* (%)	
Awareness of proper work environment and posture	
Yes, provided by workplace	98 (28%)
Yes, independently searched	107 (30%)
No	94 (26%)
Not sure	56 (16%)
Follow ergonomic work positions	
All the time	18 (5%)
Most of the time	88 (25%)
Sometimes	158 (45%)
Rarely	53 (15%)
Never	8 (2%)
Not sure	30 (8%)
Having proper information about ergonomic work positions	
Yes	139 (39%)
No	70 (20%)
Maybe	146 (41%)
Work-related injury, *n* (%)	
Yes	63 (18%)
No	235 (66%)
Not sure	57 (16%)

**Table 2:** Prevalence of MSD symptoms for different body areas in the past 12 months for each ADP occupation (*n* = 355).

Occupation	Neck	Shoulders	Upper Back	Elbows	Wrist/Hand	Lower Back	Hips/Thighs	Knees	Ankles/Feet
Dental assistant (*n* = 249)	63%	61.4%	52.6%	20.9%	52.2%	62.6%	36.1%	42.6%	48.6%
Dental lab technician (*n* = 64)	57.8%	57.8%	43.7%	15.6%	46.9%	71.9%	10.9%	37.5%	31.2%
Dental sterilization (*n* = 32)	53.1%	62.5%	56.2%	25%	56.2%	78.1%	37.5%	34.4%	62.5%
Dental radiology technician (*n* = 10)	70%	70%	30%	20%	60%	80%	20%	40%	70%

**Table 3:** Reported typical physical movement performed for ADP occupations (*n* = 355).

**Occupation**	**Standing for Long Time**	**Sitting for Long Time**	**Lifting Heavy Objects**	**Pushing Carts or Heavy Objects**	**Walking Long Distances**	**Rotation**	**Bending**	**Repetitive Hand and Wrist Movement**	**Working with and/or Operating Large Machines**	**Working withand/or Operating Machines that Produce Vibration**	**Keeping an Uncomfortable Posture for Long Periods of Time**
Dental assistant (*n* = 249)	77.5%	31.3%	19.3%	20%	30.9%	44.1%	38.1%	57.4%	17.7%	18.9%	47.8%
Dental lab technician (*n* = 64)	62.5%	51.6%	21.9%	17.1%	31.2%	50%	53.1%	64%	34.4%	46.9%	59.4%
Dental sterilization (*n* = 32)	84.4%	21.9%	43.7%	46.9%	28.1%	43.7%	56.2%	68.7%	46.9%	15.6%	50%
Dental radiology technician (*n* = 10)	80%	20%	30%	20%	10%	60%	50%	70%	20%	0%	60%
Total (All ADP) (*n*= 355)	75.2%	33.8%	22.2%	22%	3.2%	45.3%	42.5%	59.7%	23.1%	23.1%	50.4%

**Table 4:** Prevalence of Physician visits and pain medication taken for different body areas in the past 12 months for each ADP occupation (*n* = 355).

Body Area	Dental Assistant (*n* = 249)		Dental Lab Technician (*n* = 64)		Dental Sterilization (*n* = 32)		Dental Radiology Technician (*n* = 10)		Total (*n* = 355)	
	Physician Visits	Pain Medication	Physician Visits	Pain Medication	Physician Visits	Pain Medication	Physician Visits	Pain Medication	Physician Visits	Pain Medication
Neck	24.1%	30.1%	20.3%	28.3%	28.1%	43.7%	30%	40%	23.9%	31.3%
Shoulders	20%	30.1%	26.6%	28.1%	21.9%	34.4%	40%	40%	22%	30.4%
Upper back	17.7%	23.3%	18.7%	20.3%	34.4%	28.1%	40%	20%	20%	23.1%
Elbows	10.4%	8%	9.4%	4.7%	12.5%	6.2%	10%	0%	10.4%	7%
Wrist/Hands	18.5%	17.7%	15.6%	17.2%	21.9%	28.1%	30%	20%	18.6%	18.6%
Lower Back	27.3%	36.9%	21.9%	31.2%	43.7%	50%	40%	60%	28.2%	37.7%
Hips/Thighs	12.4%	10.8%	14%	9.4%	9.4%	15.6%	10%	10%	12.4%	11%
Knees	18%	18%	18.7%	12.5%	25%	18.7%	10%	30%	18.6%	17.5%
Ankles	19.7%	20%	17.2%	12.5%	28.1%	18.7%	30%	50%	20.3%	19.4%

## Data Availability

The datasets generated during and/or analysed during the current study are available from the corresponding author on reasonable request.
